# Transcriptome analysis of the response provided by *Lasiopodomys mandarinus* to severe hypoxia includes enhancing DNA repair and damage prevention

**DOI:** 10.1186/s12983-020-00356-y

**Published:** 2020-03-31

**Authors:** Qianqian Dong, Zishi Wang, Mengwan Jiang, Hong Sun, Xuqin Wang, Yangwei Li, Yifeng Zhang, Han Cheng, Yurong Chai, Tian Shao, Luye Shi, Zhenlong Wang

**Affiliations:** 1 School of Life Sciences, Zhengzhou University, Zhengzhou, 450001 Henan Province China; 2 College of Physical Education (main campus), Zhengzhou University, Zhengzhou, Henan Province China; 3 Central Laboratory, The Affiliated Cancer Hospital of Zhengzhou University, Zhengzhou, 450008 Henan Province China; 4 School of Basic Medical Sciences, Zhengzhou University, Zhengzhou, 450001 Henan Province China

**Keywords:** *Lasiopodomys mandarinus*, *Lasiopodomys brandtii*, Severe hypoxia, Immune responses, Transcriptome

## Abstract

**Background:**

Severe hypoxia induces a series of stress responses in mammals; however, subterranean rodents have evolved several adaptation mechanisms of energy metabolisms and O_2_ utilization for hypoxia. Mammalian brains show extreme aerobic metabolism. Following hypoxia exposure, mammals usually experience irreversible brain damage and can even develop serious diseases, such as hypoxic ischemic encephalopathy and brain edema. To investigate mechanisms underlying the responses of subterranean rodents to severe hypoxia, we performed a cross-species brain transcriptomic analysis using RNA sequencing and identified differentially expressed genes (DEGs) between the subterranean rodent *Lasiopodomys mandarinus* and its closely related aboveground species *L. brandtii* under severe hypoxia (5.0% O_2_, 6 h) and normoxia (20.9% O_2_, 6 h).

**Results:**

We obtained 361 million clean reads, including 69,611 unigenes in *L. mandarinus* and 69,360 in *L. brandtii*. We identified 359 and 515 DEGs by comparing the hypoxic and normoxia groups of *L. mandarinus* and *L. brandtii*, respectively. Gene Ontology (GO) analysis showed that upregulated DEGs in both species displayed similar terms in response to severe hypoxia; the main difference is that GO terms of *L. brandtii* were enriched in the immune system. However, in the downregulated DEGs, GO terms of *L. mandarinus* were enriched in cell proliferation and protein transport and those of *L. brandtii* were enriched in nuclease and hydrolase activities, particularly in terms of developmental functions. Kyoto Encyclopedia of Genes and Genomes (KEGG) pathway analysis revealed that upregulated DEGs in *L. mandarinus* were associated with DNA repair and damage prevention as well as angiogenesis and metastasis inhibition, whereas downregulated DEGs were associated with neuronal synaptic transmission and tumor-associated metabolic pathways. In *L. brandtii*, upregulated KEGG pathways were enriched in the immune, endocrine, and cardiovascular systems and particularly in cancer-related pathways, whereas downregulated DEGs were associated with environmental information processing and misregulation in cancers.

**Conclusions:**

*L. mandarinus* has evolved hypoxia adaptation by enhancing DNA repair, damage prevention, and augmenting sensing, whereas *L. brandtii* showed a higher risk of tumorigenesis and promoted innate immunity toward severe hypoxia. These results reveal the hypoxic mechanisms of *L. mandarinus* to severe hypoxia, which may provide research clues for hypoxic diseases.

## Background

Oxygen (O_2_) is essential for the growth and reproduction of aerobic organisms and is a crucial factor for the metabolism of living organisms. Typically, O_2_ concentrations in the surroundings change with temperature, humidity, atmospheric pressure, and altitude, among other factors. Many hypoxic environments occur naturally, such as the aquatic habitats, high altitudes [1], underground burrows [2], and tumor microenvironment [3, 4]. In many vertebrates, O_2_ lack for a brief period in the brain tissue can inflict irreversible neuronal damage. For instance, in pregnant guinea pigs, hypoxic conditions resulted in shrinkage of the hippocampal CA1 neurons’ apical and basal dendritic tree and a decreased number of branches and branching points in the granular cells of the dentate gyrus [5]; severe hypoxia can even cause hypoxic–ischemic encephalopathy and brain edema [6, 7]. However, hypoxia does not lead to the same damage in all living organisms. Certain species that chronically inhabit hypoxic ecological niches have developed unique and effective strategies and physiological mechanisms to survive under hypoxia [8–10].

Subterranean rodents naturally occur in enclosed and hypoxic underground burrows. They face challenges such as severe hypoxia and limited food availability during monsoon and winter when the soil freezes. As a result, rodents have evolved various strategies and characteristics to overcome these harsh environmental conditions [8]; for instance, upon the onset of monsoon, *Spalax carmeli* exhibits specific differences in blood properties [11] as well as shows high capillary density and total mitochondrial volume [12], which help them in maintaining normal foraging and burrowing activities in environments with extremely low O_2_ concentrations (7.2%) [8]. Additionally, *S. ehrenbergiana* survive for > 11 h in an extreme hypoxic environment (3% O_2_), whereas rats can only survive for up to 2.5 h in such an environment [13].

In recent years, there has been extensive research on physiological and molecular adaptive mechanisms of subterranean rodents to hypoxic environments. Compared with ground rodents, subterranean rodents have evolved unique protective mechanisms in response to hypoxic conditions. *Heterocephalus glaber* (the naked mole-rat, NMR) neurons show significant cellular resistance to acidotoxicity compared with mouse neurons, perhaps owing to reduced ASIC-mediated currents and NaV activity in NMR. Moreover, NMR neurons maintain synaptic transmission much longer than mouse neurons and can even recover after anoxia periods of over 30 min. NMRs have also evolved the ability to use fructose to fuel vital organs, such as the heart and brain, under near-anaerobic conditions [7, 14, 15]. Under hypoxia, genes related to DNA repair and metabolic pathways, which are essential to overcome dysfunctional replication and oxidative stress, were significantly upregulated in *Spalax* species but downregulated in other species [16, 17]. Additionally, the expression level of O_2_-binding respiratory proteins, such as neuroglobin and cytoglobin in *Spalax* was significantly higher than that in rats [18].

*Lasiopodomys mandarinus* is a subterranean rodent widely distributed throughout northeastern and central China, north-central Mongolia, adjacent areas of Siberia (south of Lake Baikal), and southern and central Korean Peninsula. As a subterranean species, O_2_ levels in its natural burrows can drop as low as 16.04% in summer and even further during the rainy season [19]. *L. mandarinus* exhibits remarkable physiological adaptations. Studies have shown that *L. mandarinus* shows lower hematocrit (HCT), mean corpuscular volume, mean corpuscular hemoglobin, higher mean corpuscular hemoglobin (MCHC), and capillary density than the Kunming mouse under chronic hypoxia. These changes in *L. mandarinus* decrease blood viscosity and thus resistance to blood circulation, increase O_2_ delivery and O_2_-carrying capacities and reduce diffusion distance to muscle mitochondria to maintain the high hypoxia tolerance [12, 20, 21]. Conversely, *L. brandtii*, a species closely related to *L. mandarinus*, is mainly distributed in the grasslands of middle-eastern Inner Mongolia, eastern regions of Mongolia, and some parts of southern Russia [22]. It spends most of its life above the ground. Because *L. mandarinus* and *L. brandtii* have a close evolutionary relationship and distinct life histories [23], they are ideal animal models for comparative studies on the mechanisms underlying adaptation to hypoxia in subterranean mammals [24].

Studies on hypoxia in subterranean rodents under severe hypoxia are mainly limited to blind mole and naked mole rats; further studies are needed. In the present study, we sequenced and assembled the brain transcriptomes of *L. mandarinus* and *L. brandtii* under severe hypoxia (5% O_2_) and normoxia (20.9% O_2_). Whole brain RNA was extracted and subjected to RNA sequencing (RNA-seq) to identify genes that were differentially expressed under normoxic and hypoxic conditions between the two species, with the aim to reveal the adaptive molecular mechanisms of subterranean rodents to severe hypoxic conditions.

## Results

### Illumina sequencing and de novo transcriptome assembly

We obtained 361 million reads with 91.09 billion bases following stringent quality assessment and data filtering (Tables 1 and S1). There were 137,697 transcripts (mean length: 1470.19) and 80,978 unigenes (mean length: 986.63), with an N50 (minimum contig length required to cover 50% of the genome) of 2351 for *L. mandarinus*; there were 132,622 transcripts (mean length: 1524.68) and 83,444 unigenes (mean length: 1000.63), with an N50 of 2410 for *L. brandtii*. The length distribution of the assembled unigenes is presented in Table S2.

**Table 1:** A summary of the Illumina HiSeq sequencing and assembly of *L. mandarinus* and *L. brandtii*. N50: The N50 length is used to determine the assembly continuity, the higher the better. N50 is a weighted median statistic that 50% of the total length is contained in transcripts that are equal to or larger than this value. GC (%): the percentage of G and C bases in all Unigenes

	*L. mandarinus*	*L. brandtii*
Reads length (bp)	PE; 125 bp	PE; 125 bp
Total number of raw reads	196,066,964	202,476,460
Total number of clean reads	177,508,410	184,167,383
Total Number of Transcripts	137,697	132,622
Total Number of Unigenes	80,978	83,444
N50 (All)	3097	3362
Median contig length (bp)	652	630
Average contig length (bp)	1470.19	1524.68
Assembly (bp)	202,440,402	202,206,577
N50 of Unigenes	2351	2410
Median contig length of Unigenes (bp)	403	408
Average contig length of Unigenes (bp)	986.63	1000.63
Assembly of Unigenes (bp)	79,895,482	83,496,653
GC (%)	49.13	49.28

### Functional annotation

**Fig. 1:**
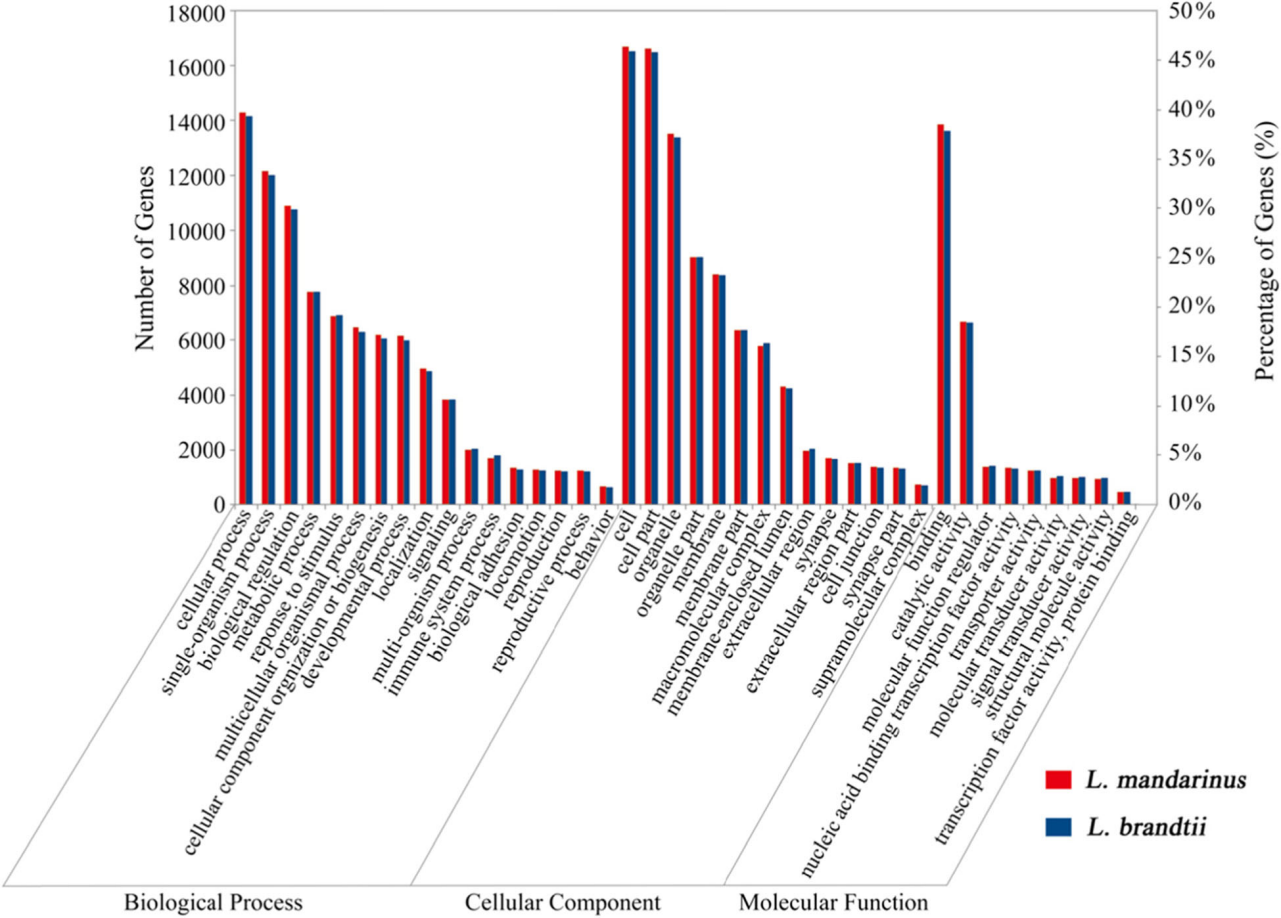
GO annotations for *L. mandarinus* and *L. brandtii* transcriptomes

According to BLASTx results, 21,012 (25.95%) unigenes of *L. mandarinus* and 20,126 (24.12%) of *L. brandtii* had homologous proteins in the National Center for Biotechnology Information (NCBI) Non-redundant (Nr) database (Table S3). Based on annotated unigenes in the database, 16,165 and 16,034 unigenes of *L. mandarinus* and *L. brandtii*, respectively, were assigned to one or more Gene Ontology (GO) terms, with 32.7 and 31.5% in cellular components, 20.6 and 20.9% in molecular functions, and 46.7 and 47.6% in biological processes, respectively (Fig. 1). To identify biological pathways that were differentially regulated between *L. mandarinus* and *L. brandtii*, the annotated unigenes were mapped to reference pathways in the Kyoto Encyclopedia of Genes and Genomes (KEGG) database. Unigenes of both *L. mandarinus* and *L. brandtii* were mapped to 368 pathways. We also searched for unigenes involved in the Eukaryotic Orthologous Group (KOG) classifications. The unigenes were subjected to functional prediction and classification using the KOG database and assigned to 25 KOG categories (Fig. S1).

### Gene expression pattern analysis

The RSEM and edgeR softwares were used to detect differentially expressed genes (DEGs) under hypoxic and normoxia conditions, with a false discovery rate (FDR) threshold of **≤**0.05 and a fold change of ≥2. In *L. mandarinus*, 1386 DEGs were identified from a total of 80,978 unigenes, of which 947 were upregulated and 439 were downregulated in the hypoxic brain relative to those in the normoxia brain (Fig. S2). Of the 1386 DEGs, 503 were annotated in at least one of the following databases: Nr (*n* = 347), NCBI Nucleotide (Nt) (*n* = 344), Swiss-Prot (*n* = 351), and KEGG (*n* = 248). Among the 347 DEGs annotated in the Nr database that were screened, 175 were upregulated, and 172 were downregulated (Table S4).

In *L. brandtii*, there were 1878 DEGs among a total of 83,444 unigenes, of which 1217 were upregulated and 661 were downregulated in the hypoxic brain relative to those in the normoxic brain (Fig. S2). Of the 1878 DEGs, 681 were annotated in at least one of the following databases: Nr (*n* = 498), Nt (*n* = 466), Swiss-Prot (*n* = 517), and KEGG (*n* = 359). Among the 498 DEGs annotated in the Nr database that were screened, 231 were upregulated, and 267 were downregulated (Table S4).

### DEG functional enrichment analysis

**Fig. 2:**
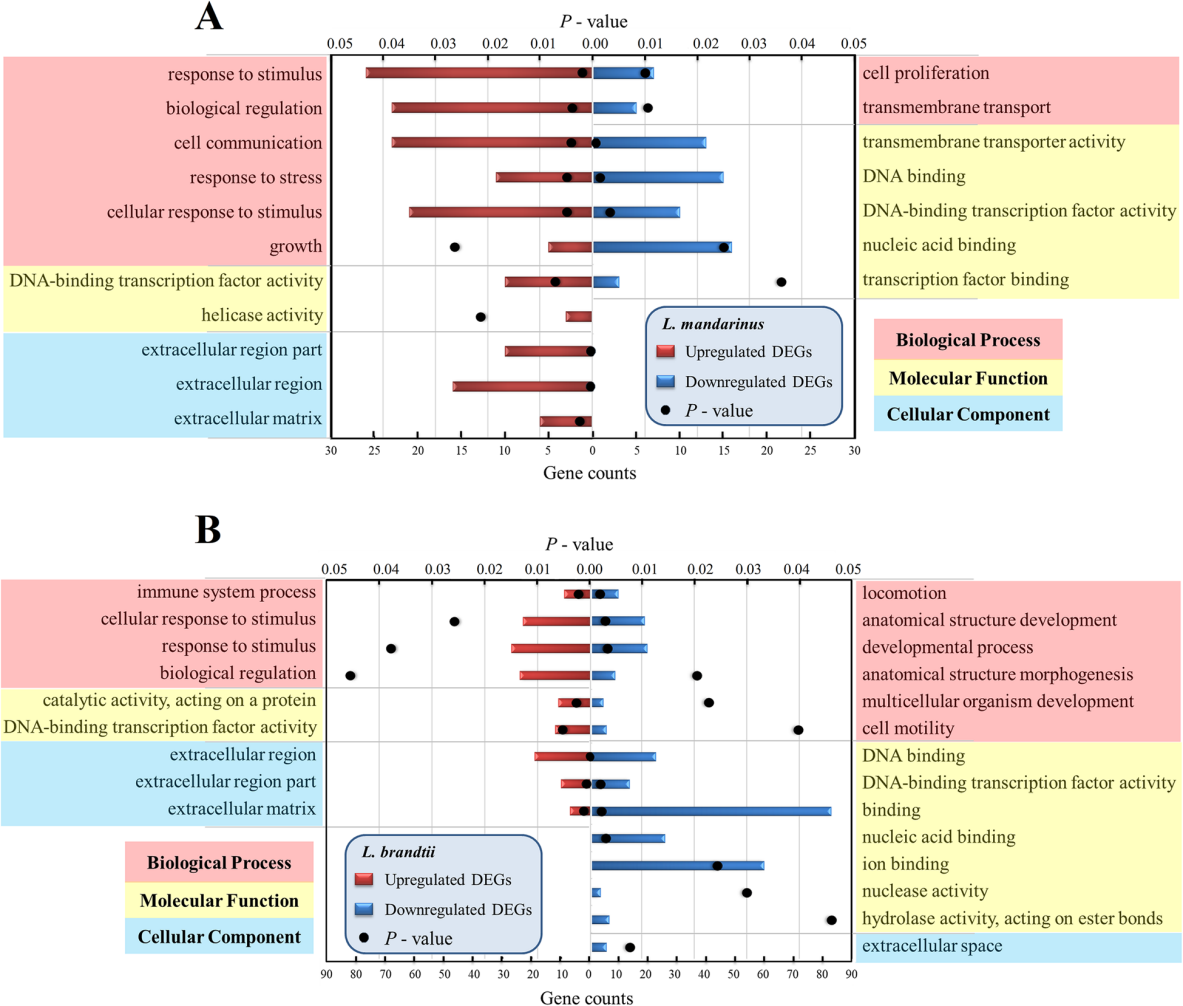
GO terms significantly enriched for up- and downregulated DEGs in *L. mandarinus* (**a**) and *L. brandtii* (**b**)

There were many GO terms enriched in the upregulated and downregulated DEGs in *L. mandarinus* and *L. brandtii*. The enriched GO terms identified in the upregulated DEGs in *L. mandarinus* have six terms for biological processes, two terms for molecular function, and three terms for cellular components. The enriched GO terms of the downregulated DEGs in *L. mandarinus* have two terms for biological processes and five terms for molecular function. For *L. brandtii*, the enriched GO terms among the upregulated DEGs have four terms for biological processes, two terms for molecular functions, and three terms for cellular components. The enriched GO terms of the downregulated DEGs mainly have six terms for biological processes and seven terms for molecular function, and only one term was enriched for cellular components (Fig. 2 and Table S5).

Many GO terms in upregulated and downregulated DEGs in *L. mandarinus* and *L. brandtii* were enriched (Fig. 2 and Table S5). The GO categories enriched for upregulated DEGs of *L. mandarinus* were mainly related to the response of cells to stimulation (GO:0050896, 0051716, and 0006950), cell signal regulation (GO:0065007 and 0007154), growth (GO:0040007), gene expression regulation (GO:0003700 and 0004386), and extracellular matrix (ECM; GO:0031012, 0044421, and 0005576), whereas most of the enriched GO terms for downregulated DEGs were related to cell proliferation (GO:0008283) and transmembrane transport (GO:0055085) as well as genes and functions related to nucleic acid binding and regulation (GO:0003677, 0003700, and 0003676) (Fig. 2a and Table S5).

Enrichment functions of upregulated DEGs in *L. brandtii* were mostly consistent with those in *L. mandarinus*. In contrast with *L. mandarinus*, the biological process function of *L. brandtii* involved the immune system (GO: 0002376) (Fig. 2b). Enrichment functions of downregulated DEGs in *L. brandtii* were different from those in *L. mandarinus*, except for three entries (GO: 0003677, 0003700, and 0003676). In particular, many development terms were enriched; the corresponding GO ontology terms included the development of anatomical structures (GO: 0048856), developmental process (GO: 0032502), anatomical morphogenesis (GO: 0009653), and development of multicellular organisms (GO: 0007275) (Fig. 2b and Table S5). Additionally, cell movement (locomotion and cell motility) as well as nuclease and hydrolase activity and binding were enriched.

### DEG pathway analysis

**Fig. 3:**
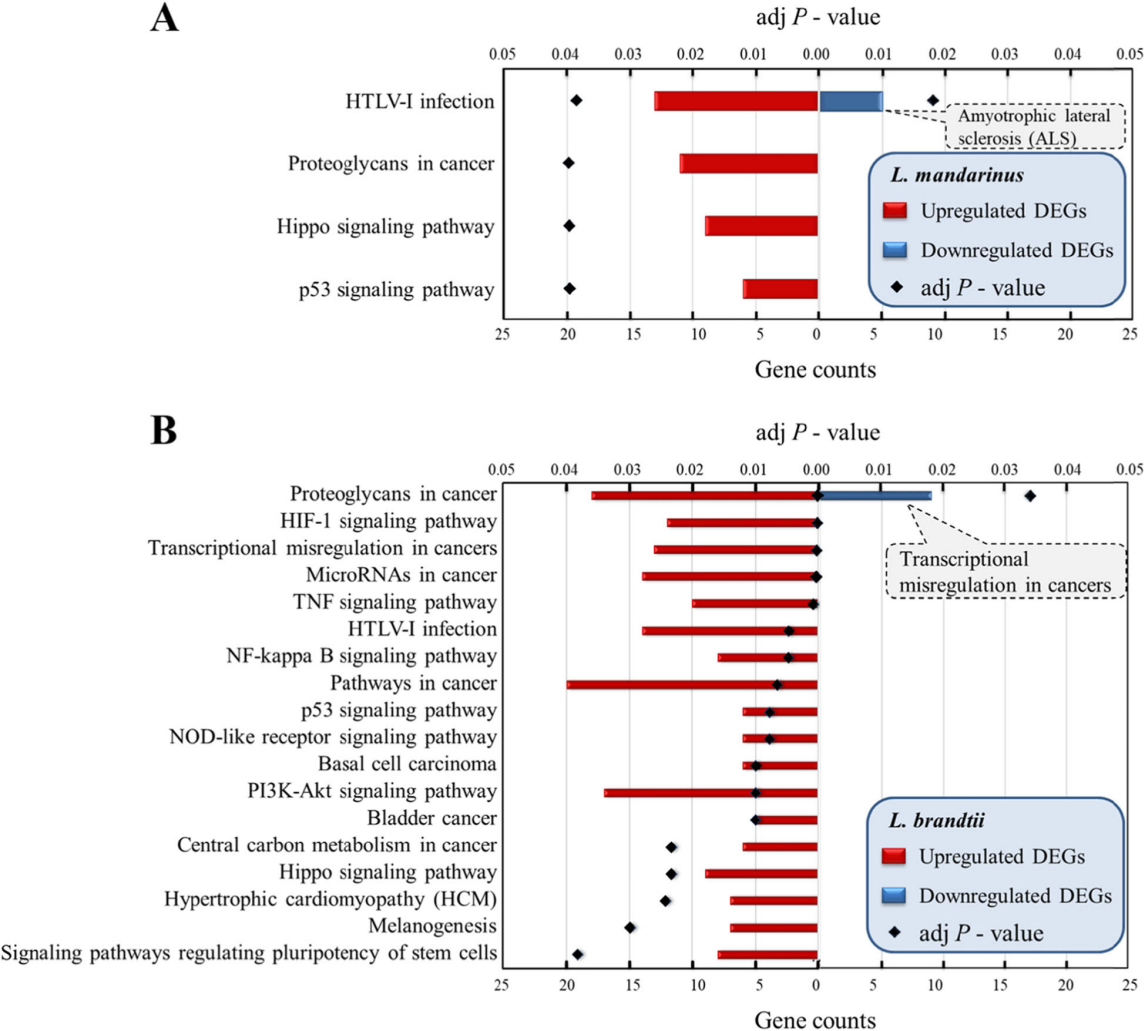
KEGG pathways significantly enriched for up- and downregulated DEGs in *L. mandarinus* (**a**) and *L. brandtii* (**b**)

To clarify the relationships among DEGs, we mapped genes in the KEGG pathway database and performed enrichment analysis using Fisher’s exact test. Pathways with more than three genes were discarded and those with both a *P*-value of < 0.05 and FDR of < 0.05 for upregulated DEGs were selected as enriched pathways. However, the enriched pathways for downregulated DEGs were no longer significant following FDR correction, although their raw data *P*-values were < 0.05. More importantly, the association with neuromodulation indicated that the genes these pathways describe might warrant further investigation. Finally, we identified four enriched pathways for upregulated and one for downregulated DEGs in *L. mandarinus* and 18 pathways for upregulated and one for downregulated DEGs in *L. brandtii* (Fig. 3 and Table S6).

Among the four enriched pathways for upregulated DEGs in *L. mandarinus*, the major functions were angiogenesis and metastasis inhibition, DNA repair and damage prevention, and cell cycle arrest and apoptosis (e.g., the p53 signaling pathway). Other pathways included key cancer-related signaling pathways studied in cancer research (e.g., HTLV-I infection, proteoglycans in cancer, and the Hippo signaling pathway). The enriched pathway for downregulated DEGs was associated with motor neuron disease (amyotrophic lateral sclerosis) (Fig. 3a and S3A and Table S6).

Four enriched pathways for upregulated DEGs in *L. brandtii* were similar to those in *L. mandarinus*. The other part of upregulated DEGs in *L. brandtii*-enriched pathways were associated with hypoxia and energy metabolism (HIF-1, NF-kappa B, and PI3K-Akt signaling pathways as well as p53 signaling pathway). DEGs, including *Angpt2* [25], *Edn1* [26]*, Nfkbia,* and *Pgf* [27] (Fig. 3b and S3B and Tables S4), in these pathways play essential roles in angiogenesis and vasoconstriction, leading to increased blood pressure. Other DEGs, including *Hk2* [28] and *Pdk1* [29] (Table S4 and Fig. S3B), in these pathways play important roles in promoting anaerobic metabolism and inhibiting tricarboxylic acid cycle metabolism*.* Notably, upregulated DEGs in *L. brandtii* were enriched in more cancer-related pathways than those in *L. mandarinus* [transcriptional misregulation in cancers; pathways associated with microRNAs in cancer; pathways in cancer; and those associated with basal cell carcinoma, bladder cancer, and central carbon metabolism in cancer] (Fig. 3b and S3B and Table S6). Moreover, pathways of upregulated DEGs in *L. brandtii* were involved in the immune, endocrine, and cardiovascular systems [e.g., NOD-like receptor signaling pathway, TNF signaling pathway, hypertrophic cardiomyopathy (HCM), and melanogenesis].

The KEGG pathways enriched for downregulated DEGs in *L. brandtii* were mainly related to transcriptional misregulation in cancers (Fig. 3b). Notably, transcriptional misregulation in cancer pathways was enriched in both upregulated and downregulated DEGs (Fig. 3b and Table S6). However, cancer types related to these upregulated and downregulated DEGs were diverse. Upregulated DEGs were mainly related to in acute myeloid leukemia, papillary renal cell carcinoma, prostate cancer, and Ewing’s sarcoma, whereas downregulated DEGs were mainly related acute lymphoblastic leukemia, neuroblastoma, and extraskeletal myxoid chondrosarcoma. This might be because some cancers readily occur under severe hypoxic conditions in *L. brandtii*.

### Validation of RNA-seq results by RT-qPCR

**Fig. 4:**
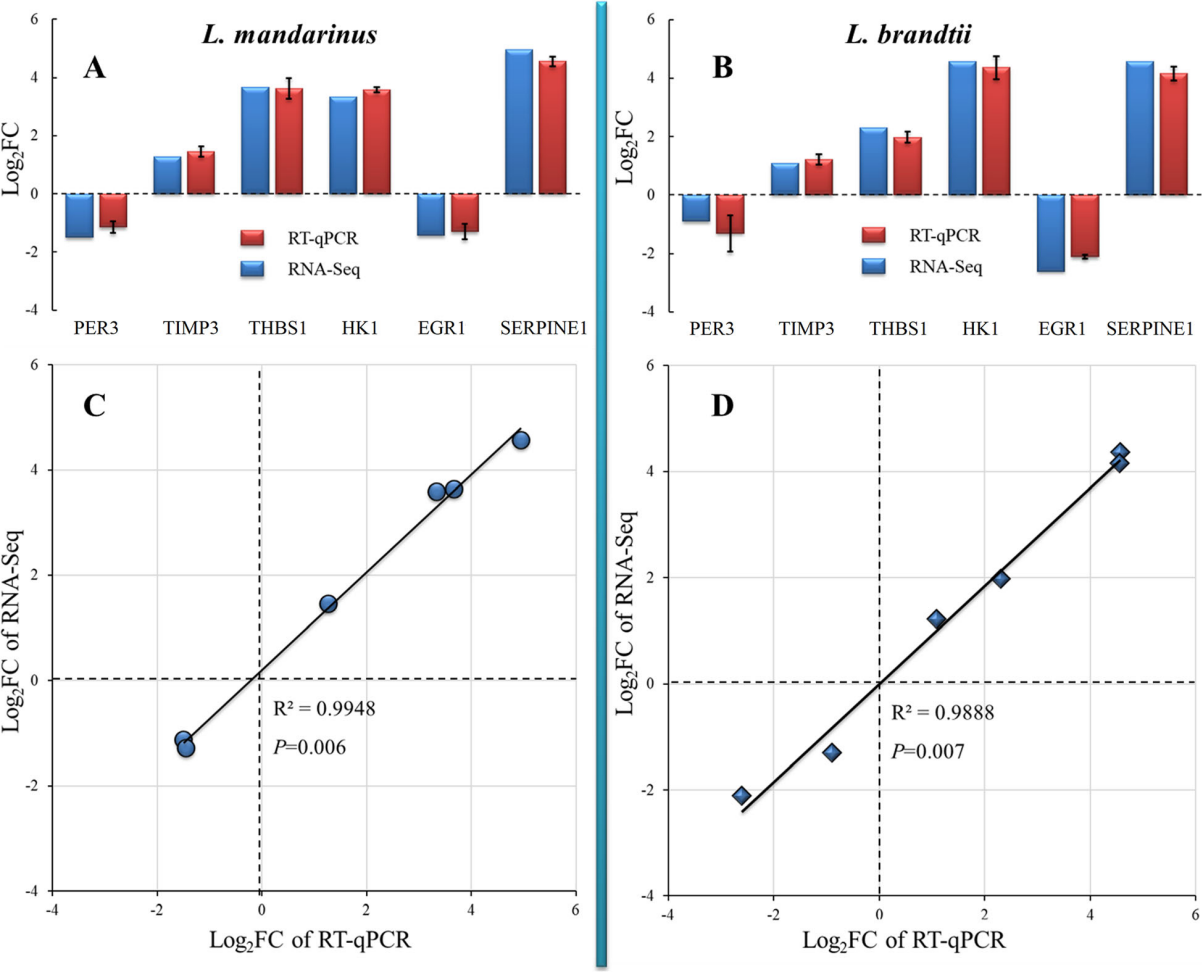
Correlations between gene expression levels measured by RT-qPCR and RNA-seq methods. The two graphs on the left side represent comparison of RNA-Seq log2FoldChange read counts with log2FoldChange RT-qPCR copy numbers (**a**) and correlations between gene expression levels (**c**) in *L. mandarinus*; the two graphs on the right side represent comparison of RNA-Seq log2FoldChange read counts with log2FoldChange RT-qPCR copy numbers (**b**) and correlations between gene expression levels (**d**) in *L. brandtii*

The concentrations and quality of RNA samples are presented in Table S7. RNA-seq results were validated by analyzing the expression of six genes involved in angiogenesis, angiogenesis inhibition, and CO_2_ damage protection (*PER3*, *TIMP3*, *THBS1*, *HK1*, *EGR1*, and *SEPRINE1*) in both *L. mandarinus* and *L. brandtii* via RT-qPCR (Table S8). Changes in expression of all six genes, as determined by RT-qPCR, correlated with RNA-seq results (*R*^*2*^ = 0. 9948, *P* = 0.006 for *L. mandarinus* and *R*^*2*^ = 0.9888, *P* = 0.007 for *L. brandtii*; Fig. 4), indicating the reliability of data obtained from our transcriptome analysis.

## Discussion

In this study, we performed RNA-seq with whole brain tissues of *L. mandarinus* and *L. brandtii* for comparative transcriptomic analysis between severe hypoxia (5% O_2_) and normoxia. We found that *L. mandarinus* possessed a unique hypoxia adaptation, which involved enhancing DNA repair and damage prevention. DEGs in *L. mandarinus* enriched in the p53 pathway can inhibit angiogenesis and metabolism, which may be related to hypoxic adaptation. *L. brandtii* showed innate immune responses and a high risk of tumorigenesis in response to severe hypoxia.

### Adaptations of *L. mandarinus* to hypoxia

**Fig. 5:**
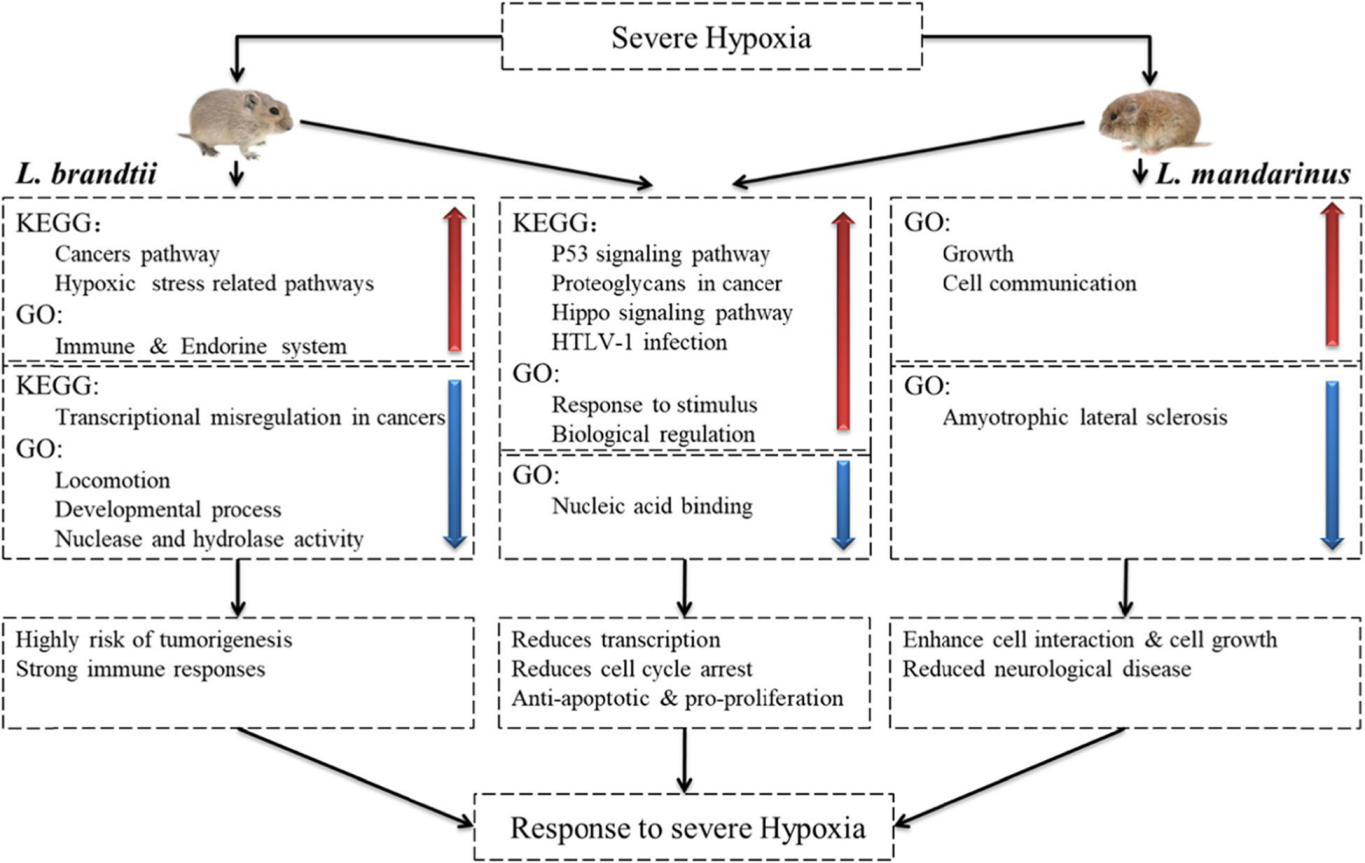
Presumed responses of *L. mandarinus* and *L. brandtii* to chronic hypoxia. The red arrows represent the KEGG pathway and GO terms enriched by upregulated DEGs. The blue arrows represent the KEGG pathway and GO terms enriched by downregulated DEGs. The black arrow represents the direction of the flow chart. The boxes corresponding to the black double arrow are similar responses of both species to hypoxia

Subterranean rodents often experience severe environment, such as rain and snowfall, in which decreased soil permeability can cause severe hypoxic conditions in underground habitats [8]. After millions and tens of millions of years, the adaptation mechanisms for hypoxia have evolved. Previous studies have shown that compared with *Rattus norvegicus*, *Spalax* can survive in lower O_2_ concentrations for significantly longer periods [13, 30]; we have found that *L. mandarinus* has a situation that similar to that of *L. brandtii* [24]. Our results revealed that although *L. mandarinus* is highly affected when exposed to a severely hypoxic environment, it showed better adaptability than *L. brandtii*. Moreover, hypoxia-activated genes in *L. mandarinus* were significantly enriched with a battery of ontologies known to be associated with angiogenesis and stress response, including ECM, cell communication, and stress response, among others (Fig. 2a and 5 and Table S5). Chemotactic signals encoded by ECM are essential to normoxia the migration and interactions of endothelial cells with supporting cells during angiogenesis [17, 31]. Under severe hypoxia, DEGs in *L. mandarinus* showed enriched growth-related functions. In general, hypoxia leads to the retardation of growth and development in mammals, thereby reducing metabolism, which is an important mechanism of hypoxia response [32]. In *Spalax*, genes involved in the growth and development terms were also upregulated under hypoxia [17]. Because both *L. mandarinus* and *Spalax* are subterranean rodents, we speculated that an upregulated expression of development-related genes reflects the normoxia of cell proliferation and differentiation during hypoxia in subterranean rodents, which is one of the common mechanisms by which they respond to severe hypoxia. Conversely, we found that *L. brandtii* showed many enriched development-related gene functions in the downregulated DEGs (Fig. 2b). We also found that downregulated DEGs enriched GO terms in *L. brandtii*, such as location and cell motility as well as nuclease and hydrolase activity and binding, indicating that DNA replication and transcriptional functions in *L. brandtii* are downregulated and cell division is inhibited under severe hypoxia. The brain tissue of *L. brandtii* is unable to carry out normal physiological and metabolic processes.

### Enhancement of immune function in *L. brandtii*

We found that *L. brandtii* showed high expression of certain genes involved in the HIF pathway but not of the *Hif* gene itself. Although HIF is expected to increase acutely after the onset of hypoxia [33], its expression gradually falls over time [34, 35] . The downstream genes and proteins, however, remain at higher than normal expression levels for a while after the HIF spike. In the HIF pathway enriched by the upregulated DEGs in *L. brandtii*, in addition to the important roles of *Hk2* and *Pdk1* in metabolism, *Angpt2* and *Serpine1* played important roles in angiogenesis [25, 36].

Upregulated DEGs in *L. brandtii* involved in melanogenesis exhibit powerful antioxidant functions [37], which are related to the endocrine system (Fig. 3a and S3B and Table S5). Moreover, other upregulated DEGs involved in melanosome functions are enriched in the muscles of *Spalax* exposed to 6% hypoxia [17]. Thus, we speculated that the genes related to melanosomes enhanced the antioxidation potential of the body, which is a common mechanism of response to severe hypoxia in rodents. When the brain is in the hypoxic condition, oxidative stress and antioxidant defense also become very relevant [38, 39]. Although we found an indirect link between antioxidant functions and hypoxia in the GO enrichment results of *L. brandtii*, there was no pathway or function that was directly related to oxidative stress and antioxidant defense based on the enrichment results of KEGG and GO.

In addition, we found that hypoxia-activated *L. mandarinus* and *L. brandtii* genes have significantly enriched GO terms with respect to ECM. In addition to being essential for the normoxia of endothelial cell (EC) migration and their interaction with supporting cells [31, 40], during angiogenesis, the ECM also modulates EC cell survival by integration of signals induced by the binding of EC integrins to the ECM with those induced by growth factors [31]. Multiple factors affect cell survival, including hypoxia and inflammation, which are known to modulate the balance between EC apoptosis and survival [17].

It is noteworthy that *L. brandtii* showed a strong immune response to severe hypoxia. As an aboveground rodent, *L. brandtii* showed a response pattern similar to that of other aerobic organisms, such as rats [16], and the number of DEGs in *L. brandtii* was much higher than that in *L. mandarinus*. Upregulated DEGs in *L. brandtii* were enriched in the NOD-like receptor signaling pathway, which plays important roles in the innate immune system (Fig. 3b and S3B). Card9 mediates signals from pattern recognition receptors to downstream signaling pathways and activates proinflammatory cytokines and anti-inflammatory cytokines via an innate immune response to clear an infection [41]. Pstpip1 also has a role in innate immunity and the inflammatory response by regulating endocytosis and cell migration in neutrophils [42]. Birc3 acts as an important regulator of innate immune signaling via the regulation of Toll-like receptors, NOD-like receptors and RIG-I like receptors [43]. Tnfaip3 has been shown to be critical for limiting inflammation by terminating endotoxin- and TNF-induced NF-kappa B responses [44]. Moreover, other upregulated DEGs were also significantly enriched in the immune system (GO: 0002376) (Fig. 2b). Protein–protein interaction analysis of common DEGs in *L. brandtii* showed that the most significant core genes were *CXCL1*, *MMP9*, *JUN*, *ANXA2*, and *F5*, which regulate immune response and cancer progression (Fig. S4A and Table S4). *CXCL1* encodes the C–X–C motif chemokine ligand 1, which is expressed by macrophages, neutrophils, and epithelial cells [45, 46], and shows neutrophil chemoattractant activity [47, 48]. *CXCL1* expression levels are strongly correlated with the occurrence of certain tumors and promotion of tumor progression through stimulating angiogenesis [49]. *MMP9* encodes matrix metallopeptidase 9, which plays important roles in angiogenesis and neovascularization and is probably involved in the development of several human malignancies [50]. Furthermore, *MMP9* expression is elevated in rheumatoid arthritis [51] and focal brain ischemia [52]. Activated *JUN* encodes a jun proto-oncogene, an AP-1 transcription factor subunit, which is associated with cancer cell proliferation and angiogenesis [53]. The annexin A2 protein encoded by *ANXA2* is a key determinant of epithelia -mesenchymal transformation of brain tumor cells, and its functions outside the cell in anticoagulant reactions have been implicated [54]. *F5* encodes the coagulation factor V, which is a central regulator of hemostasis, and its lack predisposes animals to hemorrhage. Therefore, interactions of these proteins likely result strong immune responses and high tumor risk in *L. brandtii* under severe hypoxia (Fig. 5).

Tumor surveillance of the immune system is an important barrier to cancer development. Results of a previous comparative study on mice and *Spalax* has revealed that *Spalax* had better tumor inhibition ability than mice, and this ability was speculated to be closely related to the up-regulation of immune-related functions [55]; this finding is inconsistent with ours and needs further study. We propose two reasons for this difference. First, the innate immune system is an ancient evolutionary defense strategy. Mice were model animals bred in laboratory and reared in captivity with cleaner environments than those in the underground burrows of *Spalax*. Conversely, *L. mandarinus* and *L. brandtii* are related species that live in the wild. *L. brandtii* is mainly active on the ground, living in a more complex environment and evolving more immune-related functions [56]. Meanwhile, *L. mandarinus* is a subterranean rodent that inhabits a relatively simple environment and did not evolve many immune responses. Second, *L. mandarinus* shows better DNA repair and cell protection functions under severe hypoxia than *L. brandtii* and does not need to mobilize a sizable immune response to eliminate apoptotic or necrotic cells produced under severe hypoxia. Immunization is an aerobic process. Immune response is an energy-consuming process. Severe hypoxia can lead to a lack of energy, which may reduce immune sensitivity and vulnerability and even cause disease and lead to death in animals.

Collectively, *L. mandarinus* showed a better adaptive response but a lower immune response to severe hypoxia than *L. brandtii*. Differences in gene expressions between these two voles under severe hypoxia may be attributed to their different life histories. *L. mandarinus* inhabits underground burrows system for almost all its life, whereas *L. brandtii* spends most of its life above the ground. Furthermore, it takes much less time for *L. mandarinus* to experience the hypoxic environment when surviving in the wild than for *L. brandtii* that uses underground burrows to rest. This is reflected by more DEGs and greater impairment of cerebral neuroprotection under severe hypoxia in *L. brandtii* than in *L. mandarinus*.

Moreover, *L. mandarinus* may be at a lower risk of tumorigenesis than *L. brandtii*. First, the hypoxic living environment is similar to that produced by the rapid growth of tumor cells. Many studies of subterranean rodents, such as *H. glaber* and *Spalax*, have demonstrated anticancer properties [55, 57–59]. The ability of subterranean rodents to suppress primary cancer is thought to be related to their long-term exposure to hypoxia in underground burrows [60]. Second, there were more upregulated DEGs and enriched cancer-related pathways in *L. brandtii* than in *L. mandarinus* (Fig. S3 and S6). The Hippo signaling pathway of *L. mandarinus* involves more genes that regulate anti-apoptotic and proliferative cellular processes, and strong regulation of anti-apoptotic genes has also been observed in *Spalax* under hypoxia [17]. Evolution of cancer resistance and hypoxia tolerance requires a common shift toward anti-apoptotic functions [17]. Thus, this may also be an important factor in the anti-tumor response of *L. mandarinus*. Third, protein interaction networks of unique DEGs of the two voles (Fig. S4B and Table S4) relabeled that among the severe hypoxia-specific DEGs in *L. mandarinus*, the most prominent core genes were *RLN3*, *AVP*, *CALCA*, and *SOX2*, which regulate responses to stress, metabolism, and cancer. *RLN3* encodes relaxin 3, which is mostly expressed within brain neurons and modulates arousal, response to stress, foraging and metabolism, and memory [61]. *AVP* encodes vasopressin, a peptide prohormone synthesized in hypothalamus neurons, which acts as a direct regulator of stress and immune responses [62]. *CALCA* encodes calcitonin-related peptide alpha, a potent peptide vasodilator that may modulate various physiological functions in all major systems (e.g., respiratory, endocrine, gastrointestinal, immune, and cardiovascular systems) [63]. *SOX2* encodes sex-determining region Y-box 2, which is a key regulatory factor promoting cancer progression by regulating the relevant genes [64]. Regulation of these proteins may be associated with a lower risk of tumorigenesis in *L. mandarinus* than in *L. brandtii*.

### Limitations of the study

This study has some limitations. We did not collect data of the changes of physiological indicators such as hematology of two voles under hypoxic environment. If we combine the transcriptomics data with physiological indicators, this study will become more credible. In addition, our selection of severe hypoxic treatment intensity (5% O_2_, 6 h) is mainly based on previous studies about *Spalax* by Malik et al. [16, 17]; this treatment can ensure the survival of *L. brandtii* for tissue sampling and RNA sequencing. Finally, due to the limitation of the splicing strategy of unigenes, we can only perform enrichment analysis on the DEGs of the same species in hypoxia and normoxia and cannot analyze the differences in GO and KEGG before and after hypoxia.

## Conclusions

*L. mandarinus* has evolved hypoxia adaptation by enhancing DNA repair and damage prevention. Simultaneously, augmenting sensing and regulating the expressions of angiogenesis- and tumor-associated genes may also play an important role in its hypoxia adaptation. However, *L. brandtii* showed a higher risk of tumorigenesis and promoted innate immunity functions, such as proinflammatory cytokine processing and neutrophil recruitment in response to severe hypoxia. These results reveal the hypoxic mechanisms of *L. mandarinus* to severe hypoxia, which may provide research clues for hypoxic diseases.

## Methods

### Animals and hypoxia treatment

We captured live *L. mandarinus* from a cropland in Xinzheng, Henan, China (34°52′ N, 113°85′ E) and obtained *L. brandtii* from the Chinese Academy of Agricultural Science. The animals were housed in laboratory at the School of Life Sciences in Zhengzhou University, China. Both species that were used in this study were offspring raised in the laboratory for at least one generation and showed a similar weight (40 ± 5 g). The animals were transferred to individual polycarbonate cages (37 × 26 × 17 cm^3^) and maintained for at least 2 weeks following which they were maintained at 20 °C–24 °C under a long-day [14-h light:10-h dark cycle (14 L:10D)] photoperiod in a laboratory suitable for reproduction [65, 66]. Specific data are shown in Table S9.

To mimic hypoxia, 12 healthy adult male voles (12 weeks of age, *n* = 6 of each species, Table S9) were randomly divided into either the severe hypoxia (5% O_2_ for 6 h) or the normoxia (20.9% O_2_) group. A DS-II hyperbaric cabin (Huaxin Hyperbaric Cabin, Weifang, China) was used to simulate severe hypoxia, and an oximeter was used to monitor O_2_ levels in the cabin. An O_2_ analyzer (Talantek, Beijing, China) was used to measure O_2_ level in the DS-II hyperbaric cabin to maintain it at 5%. A constant O_2_ level was maintained by balancing the flow rate of O_2_ and N_2_ and placing a bottle containing sodium hydroxide in the cabin to absorb CO_2_ released by the animals_._ Both *L. mandarinus* and *L. brandtii* were immediately sacrificed with an overdose of pentobarbital sodium following hypoxia treatment. Their brains were immediately harvested, flash-frozen in liquid nitrogen, and stored at − 80 °C until use.

### RNA extraction, cDNA library preparation, and RNA-seq

Whole brain tissue of each sample was individually ground in liquid nitrogen and then sampled to extract total RNA. Samples were shipped on dry ice to Biomarker Technologies Corp. (Beijing, China) for RNA-seq. Briefly, after extracting total RNA from each sample using the TRIzol reagent (Invitrogen, Carlsbad, CA, USA), the extracted RNA was treated with RNase-free DNase I (Takara Bio, Dalian, China) to remove residual DNA. Agarose gel electrophoresis (1.2%) was performed to verify RNA integrity, and Agilent 2100 Bioanalyzer (Agilent Technologies, Santa Clara, CA, USA) was used to measure RNA concentration. RNA purity was checked using the NanoPhotometer® spectrophotometer (IMPLEN, CA, USA). High-quality RNA samples were selected for cDNA library construction and sequencing. Briefly, TruSeq RNA Sample Prep Kits (Illumina, San Diego, CA, USA) were used to generate RNA-seq libraries. QIAQuick PCR Purification Kits (Qiagen, Hilden, Germany) were used to purify cDNA. cDNA libraries were constructed using inserts averaging 250 bp (range, 150–250 bp) by non-stranded library preparation. Sequencing was performed via a paired-end 125-cycle rapid run on an Illumina HiSeq 2500 System.

### Read filtering and sequence assembly

High-quality clean reads were obtained by removing adaptor sequences, duplicated sequences, and ambiguous (“N”) and low-quality reads using the Trimmomatic read trimming tool and SOAPnuke [67, 68]. Transcriptomes of the two species were separately assembled de novo using the short-reads assembly program Trinity [69]. After assembly, the TIGR Gene Indices tool was used to cluster and remove redundant transcripts [70]. After removing redundancies, the longest transcripts were considered as unigenes and subjected to downstream functional annotation and coding sequence (CDS) prediction [71].

### Functional annotation

Unigenes of *L. mandarinus* and *L. brandtii* were compared using BLASTx against the Nr [72], KEGG [73], GO [74], KOG [75], and Swiss-Prot [76] databases (e-value ≤1e^− 5^) to retrieve protein functional annotations based on sequence similarity. Gene names were assigned based on the best BLAST hit. High-priority databases (followed by Nr, Swiss-Prot, and KEGG) were selected to determine the direction of unigene sequences. Sequences showing the best alignment were used to predict CDSs. TransDecoder-v5.5.0 (Find Coding Regions within Transcripts) (https://github.com/TransDecoder/TransDecoder/releases) was used to identify candidate coding regions within transcript sequences. GO terms were assigned to each sequence using Blast2GO, with an e-value threshold of 1e^− 6^ for further functional categorization [77]. GO covers the following three parts: Cellular Component includes parts of a cell or its extracellular environment; Molecular Function includes the elemental activities of a gene product at the molecular level, such as binding or catalysis; Biological Process includes operations or sets of molecular events with a defined beginning and end, pertinent to the functioning of integrated living units, such as cells, tissues, organs, and organisms. Distribution of the GO functional classifications of unigenes was plotted using OmicShare (www.omicshare.com/tools). BinGO in Cytoscape [78] was used for GO enrichment analysis. KOBAS 3.0 (http://kobas.cbi.pku.edu.cn/) was used for KEGG annotation and pathway enrichment analysis of unigenes.

### DEG identification

The fragments per kilobase of exon per million mapped fragments (FPKM) method eliminated the influence of different gene lengths and sequencing levels on the calculation of gene expression. FPKM values were directly used to compare gene expression differences between the samples. The edgeR package (http://bioconductor.org/packages/release/bioc/html /edgeR.html) was used to obtain the base mean value for identifying the DEGs. To correct for multiple testing, FDR was calculated to adjust the *P*-value threshold. Transcripts with an FDR of ≤0.05 and a minimum two-fold difference in expression (|log_2_ ratio| ≥1) were considered as thresholds for the significance of gene expression differences between two groups. Additionally, information for DEGs was collected from unigene annotations, and these genes were subjected to GO and KEGG significant enrichment analyses to identify biological functions and metabolic pathways involving these genes.

### Validation of RNA-seq results by RT-qPCR

To validate the reliability of DEGs identified by RNA-seq, mRNA expression levels of six selected genes were measured by RT-qPCR using the same samples. Primers were designed using Primer-BLAST, and their sequences are shown in Table S8. All primer sets yielded a single peak in the dissociation curves, with an amplification efficiency of ~ 1.0. Three technical replicates were prepared for each gene in 96-well plates, and samples were amplified using LightCycler® 480 Instrument II (Roche Diagnostics, Mannheim, Germany). Relative gene expression levels were normalized to internal reference expression (β-actin gene) and calculated according to the 2^−ΔΔCt^ method. Correlation analysis was performed using SPSS 19.0 (IBM Corp., Armonk, NY, USA) to evaluate the concordance between RT-qPCR results and RNA-seq data. Significant *t*-test differences were defined as *P* < 0.05 and highly significant as *P* < 0.01.

## Supplementary information

**Additional file 1: Table S1.** Illumina sequencing data of the analyzed samples. **Table S2.** Length distribution and quality metrics of unigenes in *L. mandarinus* and *L. brandtii*. **Table S3.** Functional annotation results for *L. mandarinus* and *L. brandtii* transcriptomes. **Table S4.** Annotated DEGs for *L. mandarinus* and *L. brandtii*. **Table S5.** Significantly enriched GO terms for up- and downregulated DEGs in *L. mandarinus* and *L. brandtii* under severe hypoxia. **Table S6.** Enriched KEGG pathways for up- and downregulated DEGs in *L. mandarinus* and *L. brandtii* under severe hypoxia. **Table S7.** Results of RNA extraction from *L. mandarinus* and *L. brandtii* brain tissue. **Table S8.** RT-qPCR primers for the validation of RNA-Seq data. **Table S9.** Characteristics of *L. mandarinus* and *L.brandtii* samples.

**Additional file 2: Figure S1.** Classification of annotated transcriptome in the Eukaryotic Orthologous Group (KOG). A to Z represent the specific entry information of the KOG annotated by unigenes.

**Additional file 3: Figure S2.** DEGs in the brains of *L. mandarinus* and *L. brandtii* under severe hypoxia vs. normoxia. FC, fold change; FDR, false discovery rate. Red, blue, and green dots represent up- and downregulated and unchanged genes, respectively.

**Additional file 4: Figure S3.** Gene-pathway networks for DEGs in *L. mandarinus* (A) and *L. brandtii* (B) under severe hypoxia determined using the Clue GO tool in Cytoscape. Small and large dots represent DEGs and enriched pathways, respectively. Genes indicated in blue are upregulated. Lines between dots represent connections between the genes and the pathways in the network; genes with more connections are more important in the network.

**Additional file 5: Figure S4.** Protein interaction network for specific DEGs in *L. brandtii* (A) and *L. mandarinus*(B) brain under hypoxia. Pink squares represent upregulated proteins, and green squares represent downregulated proteins; size of the squares represents the importance of the protein in the network, with bigger squares indicating a greater importance; the thickness of line between the squares represents the strength of the association between two proteins, with a wider line indicating a stronger correlation.

## Data Availability

Raw Illumina sequences were deposited in the National Center for Biotechnology. Information (NCBI) database and our sequence read archive (SRA) records will be accessible via the following links after the indicated release date: https://dataview.ncbi.nlm.nih.gov/object/ PRJNA580223?reviewer=a8vqhpunmrrkao2tc4h356a01, SRA accession: SRP227339; Temporary Submission ID: SUB6478367 (*L. brandtii* under hypoxia); https://www.ncbi.nlm.nih.gov/bioproject /PRJNA543699, SRA accession: SRP198871; and Temporary Submission ID: SUB5628278 (*L. mandarinus* under hypoxia). The other raw read files are at NCBI SRA under run accession SRR7662995, SRR7662996, and SRR7662997 (*L. mandarinus’*s brain transcriptome sequencing under normoxia) and accession SRR7662993, SRR7662994, and SRR7663001 (*L. brandtii*’s brain transcriptome sequencing under normoxia).

## Abbreviations

CDS: Coding sequence
DEGs: Differentially expressed genes
ECM: Extracellular matrix
FDR: False discovery rate
FPKM: Fragments per kilobase of exon per million mapped fragments
GO: Gene Ontology
KEGG: Kyoto Encyclopedia of Genes and Genomes
KOG: Eukaryotic Orthologous Groups
NCBI: National Center for Biotechnology Information
Nr: Non-redundant
Nt: Nucleotide
RNA-seq: RNA sequencing
RT-qPCR: Quantitative reverse transcription-polymerase chain reaction
TNF: Tumor necrosis factor
