# Supplementary material for: Transcriptome analysis of the response provided by Lasiopodomys mandarinus to severe hypoxia includes enhancing DNA repair and damage prevention
Source: Front Zool. 2020 Mar 31;17:9. doi: 10.1186/s12983-020-00356-y (PMC7106638; doi:10.1186/s12983-020-00356-y)
Supplement: Supplementary file 1 — Additional file 1: Table S1. Illumina sequencing data of the analyzed samples. Table S2. Length distribution and quality metrics of unigenes in L. mandarinus and L. brandtii. Table S3. Functional annotation results for L. mandarinus and L. brandtii transcriptomes. Table S4. Annotated DEGs for L. mandarinus and L. brandtii. Table S5. Significantly enriched GO terms for up- and downregulated DEGs in L. mandarinus and L. brandtii under severe hypoxia. Table S6. Enriched KEGG pathways for up- and downregulated DEGs in L. mandarinus and L. brandtii under severe hypoxia. Table S7. Results of RNA extraction from L. mandarinus and L. brandtii brain tissue. Table S8. RT-qPCR primers for the validation of RNA-Seq data. Table S9. Characteristics of L. mandarinus and L.brandtii samples. [file 12983_2020_356_MOESM1_ESM.docx]

**Table S1.** Illumina sequencing data for analyzed samples

| Samples | Raw Reads Number | Clean Reads Number | Base Number (Gbp) | GC Content (%) | %≥Q30 |
| --- | --- | --- | --- | --- | --- |
| CK1-LM | 28,737,984 | 26,102,782 | 6.57 | 48.81% | 91.71% |
| CK2-LM | 28,679,082 | 26,042,739 | 6.56 | 49.01% | 91.31% |
| CK3-LM | 34,655,748 | 31,035,722 | 7.81 | 49.14% | 91.03% |
| Ahyp1-LM | 34,186,302 | 30,998,199 | 7.81 | 49.38% | 91.60% |
| Ahyp2-LM | 34,271,893 | 31,245,688 | 7.87 | 49.17% | 91.81% |
| Ahyp3-LM | 35,535,955 | 32,083,280 | 8.08 | 49.48% | 91.33% |
| CK1-LB | 39,107,546 | 35,420,510 | 8.92 | 50.01% | 91.43% |
| CK2-LB | 35,050,234 | 32,039,776 | 8.07 | 49.46% | 91.43% |
| CK3-LB | 32,946,493 | 29,979,937 | 7.55 | 49.15% | 91.34% |
| A-hyp1-LB | 32,069,055 | 29,343,366 | 7.39 | 49.53% | 91.70% |
| A-hyp2-LB | 31,701,790 | 28,665,533 | 7.22 | 49.76% | 90.39% |
| A-hyp3-LB | 31,601,342 | 28,718,261 | 7.24 | 49.36% | 91.69% |
| Total | 398,543,424 | 361,675,793 | 91.09 |  |  |

Notes: **Samples**: Sample name; **Raw Reads** **Number**: The reads amount before filtering; **Clean Reads Number**: The reads amount after filtering; **Clean Bases Number**: The total base amount after filtering; **GC Content (%)**: the percentage of G and C bases in all Clean reads; **%≥Q30**: The rate of bases which quality is greater than 30 value in clean reads.

**Table S2.** Length distribution and quality metrics of unigenes from the *L. mandarinus* and *L.* *brandtii*

| Length Range (bp) | *L. mandarinus* | *L.* *brandtii* |
| --- | --- | --- |
| 200- 300 | 31,952 | 32,598 |
| 300- 500 | 18,365 | 19,543 |
| 500-1000 | 12,354 | 12,932 |
| 1000-2000 | 8,782 | 8,620 |
| >2000 | 9,525 | 9,751 |
| Total Number | 80,978 | 83,444 |

Notes: **Length Range:** Length distribution of assembled unigenes; **Total Number:** The total number of Unigenes;

**Table S3.** Functional annotation results for *L. mandarinus* and *L.* *brandtii* transcriptomes

| Database | *L. mandarinus* | *L.* *brandtii* |
| --- | --- | --- |
| GO | 16165 | 16034 |
| KEGG | 12780 | 12458 |
| KOG | 13105 | 12993 |
| Swissprot | 16700 | 17084 |
| Nr | 21012 | 20126 |
| All | 25316 | 25950 |

**Table S4.** Annotated DEGs for *L. mandarinus* and *L.* *brandtii*

**Table S5.** GO terms significant enriched for up- and downregulated DEGs in *L. mandarinus* and *L.* *brandtii*

| ***L. mandarinus*** | | | | | ***L. brandtii*** | | | |
| --- | --- | --- | --- | --- | --- | --- | --- | --- |
| **Up-regulated** | | | | | **Up-regulated** | | | |
| **GO terms** | | **Ontology** | **GO ID** | ***P* value** | **GO terms** | **Ontology** | **GO ID** | ***P* value** |
| Extracellular region | | CC | 0005576 | 0.000 | Extracellular region part | CC | 0044421 | 0.000 |
| Extracellular region part | | CC | 0044421 | 0.000 | Extracellular region | CC | 0005576 | 0.000 |
| Extracellular matrix | | CC | 0031012 | 0.003 | Extracellular matrix | CC | 0031012 | 0.001 |
| Response to stimulus | | BP | 0050896 | 0.001 | Immune system process | BP | 0002376 | 0.002 |
| Biological regulation | | BP | 0065007 | 0.001 | Cellular response to stimulus | BP | 0051716 | 0.025 |
| Cell communication | | BP | 0007154 | 0.002 | Response to stimulus | BP | 0050896 | 0.037 |
| Cellular response to stimulus | | BP | 0051716 | 0.002 | Biological regulation | BP | 0065007 | 0.045 |
| Response to stress | | BP | 0006950 | 0.004 | Catalytic activity, acting on aprotein | MF | 0140096 | 0.002 |
| Growth | | BP | 0040007 | 0.026 | DNA-binding transcription factor activity | MF | 0003700 | 0.005 |
| DNA-binding transcription  factor activity | | MF | 0001071 | 0.013 |  |  |  |  |
| Helicase activity | | MF | 0004386 | 0.027 |  |  |  |  |
| **Down-regulated** | | | | | **Down-regulated** | | | |
| Cell proliferation | BP | | 0008283 | 0.013 | Locomotion | BP | 0040011 | 0.002 |
| Transmembrane transport | BP | | 0055085 | 0.013 | Anatomical structure development | BP | 0048856 | 0.003 |
| DNA binding | MF | | 0003677 | 0.001 | Developmental process | BP | 0032502 | 0.003 |
| Transmembrane transporter activity | MF | | 0022857 | 0.001 | Anatomical structure morphogenesis | BP | 0009653 | 0.020 |
| Nucleic acid binding | MF | | 0003676 | 0.001 | Multicellular organism development | BP | 0007275 | 0.023 |
| Transcription factor binding | MF | | 0008134 | 0.036 | Cell motility | BP | 0048870 | 0.039 |
| DNA-binding transcription factor activity | MF | | 0003700 | 0.003 | DNA-binding transcription factor activity | MF | 0003700 | 0.002 |
|  |  | |  |  | DNA binding | MF | 0003677 | 0.000 |
|  |  | |  |  | Binding | MF | 0005488 | 0.002 |
|  |  | |  |  | Nucleic acid binding | MF | 0003676 | 0.003 |
|  |  | |  |  | Ion binding | MF | 0043167 | 0.024 |
|  |  | |  |  | Nuclease activity | MF | 0004518 | 0.030 |
|  |  | |  |  | Hydrolase activity, acting on ester bonds | MF | 0016788 | 0.046 |
|  |  | |  |  | Extracellular space | CC | 0005615 | 0.007 |

Notes: Terms with *P* < 0.05 are shown, with a Benjamini-Hochberg correction or false discovery rate. BP: biological process; CC: cellular component; MF: molecular function.

**Table S6.** KEGG pathways enriched for up- and downregulated DEGs in *L. mandarinus* and *L.* *brandtii* under acute hypoxia

| ***L. mandarinus*** | | | ***L. brandtii*** | | |
| --- | --- | --- | --- | --- | --- |
| **Up-regulated** | | | **Up-regulated** | | |
| **Pathway** | **Map ID** | ***adj.P value*** | **Pathway** | **Map ID** | ***adj.P value*** |
| HTLV-I infection | 05166 | 0.038 | Proteoglycans in cancer | 05205 | 0.000 |
| Proteoglycans in cancer | 05205 | 0.040 | HIF-1 signaling pathway | 04066 | 0.000 |
| Hippo signaling pathway | 04390 | 0.040 | Transcriptional misregulation in cancers | 05202 | 0.000 |
| p53 signaling pathway | 04115 | 0.040 | MicroRNAs in cancer | 05206 | 0.000 |
|  |  |  | TNF signaling pathway | 04668 | 0.000 |
|  |  |  | HTLV-I infection | 05166 | 0.004 |
|  |  |  | NF-kappa B signaling pathway | 04064 | 0.004 |
|  |  |  | Pathways in cancer | 05200 | 0.007 |
|  |  |  | p53 signaling pathway | 04115 | 0.008 |
|  |  |  | NOD-like receptor signaling pathway | 04621 | 0.007 |
|  |  |  | Basal cell carcinoma | 05217 | 0.010 |
|  |  |  | PI3K-Akt signaling pathway | 04151 | 0.010 |
|  |  |  | Bladder cancer | 05219 | 0.010 |
|  |  |  | Central carbon metabolism in cancer | 05230 | 0.023 |
|  |  |  | Hippo signaling pathway | 04390 | 0.023 |
|  |  |  | Hypertrophic cardiomyopathy (HCM) | 05410 | 0.024 |
|  |  |  | Melanogenesis | 04916 | 0.030 |
|  |  |  | Signaling pathways regulating pluripotency of stem cells | 04550 | 0.038 |
| **Down-regulated** | | | **Down-regulated** | | |
| Amyotrophic lateral sclerosis (ALS) | 05014 | 0.018 | Transcriptional misregulation in cancers | 05202 | 0.034 |

Notes: Pathways with *adj.P value* < 0.05 are shown, with a Benjamini-Hochberg correction or false discovery rate.

**Table S7**. Results of RNA extraction from *L. mandarinus* and *L. brandtii* brain tissue

| Sample | RNA Concentration (ng/μl) | OD260/280 | OD260/230 | 28S/18S | RNA Integrity Number  (RIN) |
| --- | --- | --- | --- | --- | --- |
| M1 | 398 | 2.010 | 2.095 | 1.4 | 9.2 |
| M2 | 556 | 2.059 | 2.000 | 1.2 | 9.1 |
| M3 | 498 | 2.041 | 1.886 | 1.1 | 7.7 |
| M4 | 622 | 2.033 | 2.073 | 1.2 | 8.9 |
| M5 | 616 | 2.026 | 2.110 | 1.4 | 9.2 |
| M6 | 612 | 2.068 | 2.155 | 1.2 | 9.0 |
| B1 | 934 | 2.066 | 1.639 | 1.2 | 8.9 |
| B2 | 516 | 2.048 | 1.623 | 1.2 | 8.9 |
| B3 | 412 | 2.124 | 2.124 | 1.2 | 8.8 |
| B4 | 540 | 2.045 | 2.143 | 1.4 | 8.9 |
| B5 | 674 | 2.055 | 1.926 | 1.3 | 8.8 |
| B6 | 834 | 2.085 | 2.183 | 1.4 | 9.1 |

Notes: M: *L. mandarinus*; B: *L. brandtii*; The numbers 1, 2, 3 represent samples from the control group, and the numbers 4, 5, and 6 represent samples from the acute hypoxic treatment group.

**Table S8.** RT-qPCR primers for validation of RNA-Seq data

| Gene | Primer-Forward | Primer-Reverse | Efficiency |
| --- | --- | --- | --- |
| PER3 | GCATTGCAGGCAAGACTGAG | CAAGACCAGTATGCAGGAGT | 1.28 |
| TIMP3 | GCAAGGACCTCAATTACCG | AGGCGTAGTGTATGGACTGATA | 0.98 |
| THBS1 | GGAGATAACGGTGTGTTTG | CGGAGATCAGGTTGGCAT | 1.13 |
| HK1 | GAGTCTGAGGTCTACGACACC | CCCACGGGTAATTTCTTGTCC | 1.24 |
| EGR1 | GGCCCCTGGTGCTACATAATG | AGGAAGCTACAAATCGGGCAT | 1.08 |
| SERPINE1 | AGCAAGCGGGAAGAAGAGTC | CAGGGTGAGGCAAGCTAGTG | 0.88 |
| *β*-actin | GTCGTACCACTGGCATTGTG | CCATCTCTTGCTCGAAGTCC | 1.05 |

**Table S9.** Characteristics of *L. mandarinus* and *L. brandtii* samples

| Sample | Age/weeks | Gender | Body length/cm | Weight/g | Brain weight/g |
| --- | --- | --- | --- | --- | --- |
| M1 | 12 | Male | 9.5 | 40.47 | 0.5203 |
| M2 | 12 | Male | 8.5 | 40.29 | 0.5135 |
| M3 | 12 | Male | 9.0 | 41.72 | 0.5278 |
| M4 | 12 | Male | 8.9 | 43.21 | 0.5425 |
| M5 | 12 | Male | 8.2 | 40.92 | 0.5267 |
| M6 | 12 | Male | 7.9 | 40.27 | 0.5262 |
| B1 | 12 | Male | 8.4 | 42.41 | 0.5531 |
| B2 | 12 | Male | 8.9 | 40.35 | 0.5141 |
| B3 | 12 | Male | 8.0 | 43.48 | 0.5215 |
| B4 | 12 | Male | 9.8 | 40.75 | 0.5112 |
| B5 | 12 | Male | 8.4 | 43.12 | 0.5214 |
| B6 | 12 | Male | 8.9 | 43.39 | 0.5177 |

Notes: M: *L. mandarinus*; B: *L. brandtii*; The numbers 1, 2, 3 represent samples from the control group, and the numbers 4, 5, and 6 represent samples from the severe hypoxic treatment group.
