# Supplementary material for: Transcriptome analysis of the response provided by Lasiopodomys mandarinus to severe hypoxia includes enhancing DNA repair and damage prevention
Source: Front Zool. 2020 Mar 31;17:9. doi: 10.1186/s12983-020-00356-y (PMC7106638; doi:10.1186/s12983-020-00356-y)
Supplement: Supplementary file 2 — Additional file 2: Figure S1. Classification of annotated transcriptome in the Eukaryotic Orthologous Group (KOG). A to Z represent the specific entry information of the KOG annotated by unigenes. [file 12983_2020_356_MOESM2_ESM.docx]

**Figure S1.** Classification of annotated transcriptome in the Eukaryotic Orthologous Group (KOG). A to Z represent the specific entry information of the KOG annotated by unigenes.

**
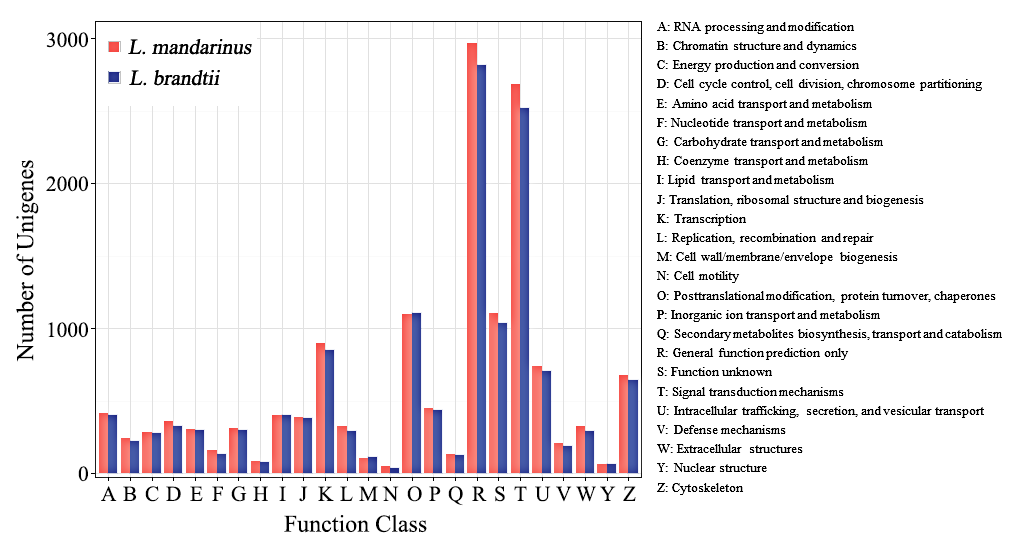
**
