# Supplementary material for: Transcriptome analysis of the response provided by Lasiopodomys mandarinus to severe hypoxia includes enhancing DNA repair and damage prevention
Source: Front Zool. 2020 Mar 31;17:9. doi: 10.1186/s12983-020-00356-y (PMC7106638; doi:10.1186/s12983-020-00356-y)

**Figure S2.** DEGs in the brain of *L. mandarinus* and *L.* *brandtii* under chronic hypoxia vs. normoxia. FC, fold change; FDR, false discovery rate. Red, blue, and green dots represent up- and downregulated and unchanged genes, respectively.


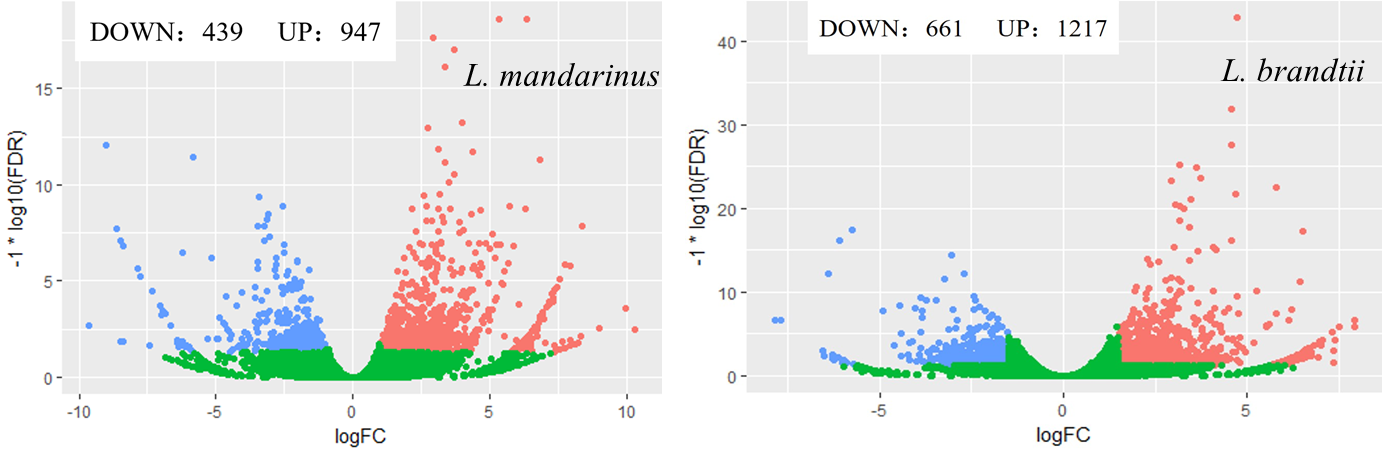

Supplement: Supplementary file 3 — Additional file 3: Figure S2. DEGs in the brains of L. mandarinus and L. brandtii under severe hypoxia vs. normoxia. FC, fold change; FDR, false discovery rate. Red, blue, and green dots represent up- and downregulated and unchanged genes, respectively. [file 12983_2020_356_MOESM3_ESM.docx]
