# Supplementary material for: Transcriptome analysis of the response provided by Lasiopodomys mandarinus to severe hypoxia includes enhancing DNA repair and damage prevention
Source: Front Zool. 2020 Mar 31;17:9. doi: 10.1186/s12983-020-00356-y (PMC7106638; doi:10.1186/s12983-020-00356-y)

**Figure S3.** Gene-pathway networks for DEGs in (A) *L. mandarinus* and (B) *L. brandtii* under severe hypoxia determined using the Clue GO tool in Cytoscape. Small and large dots represent DEGs and enriched pathways for DEGs, respectively. Genes indicated in blue are upregulated. Lines between dots represent connections between the genes and the pathways in the network; genes with more connections are more important in the network.


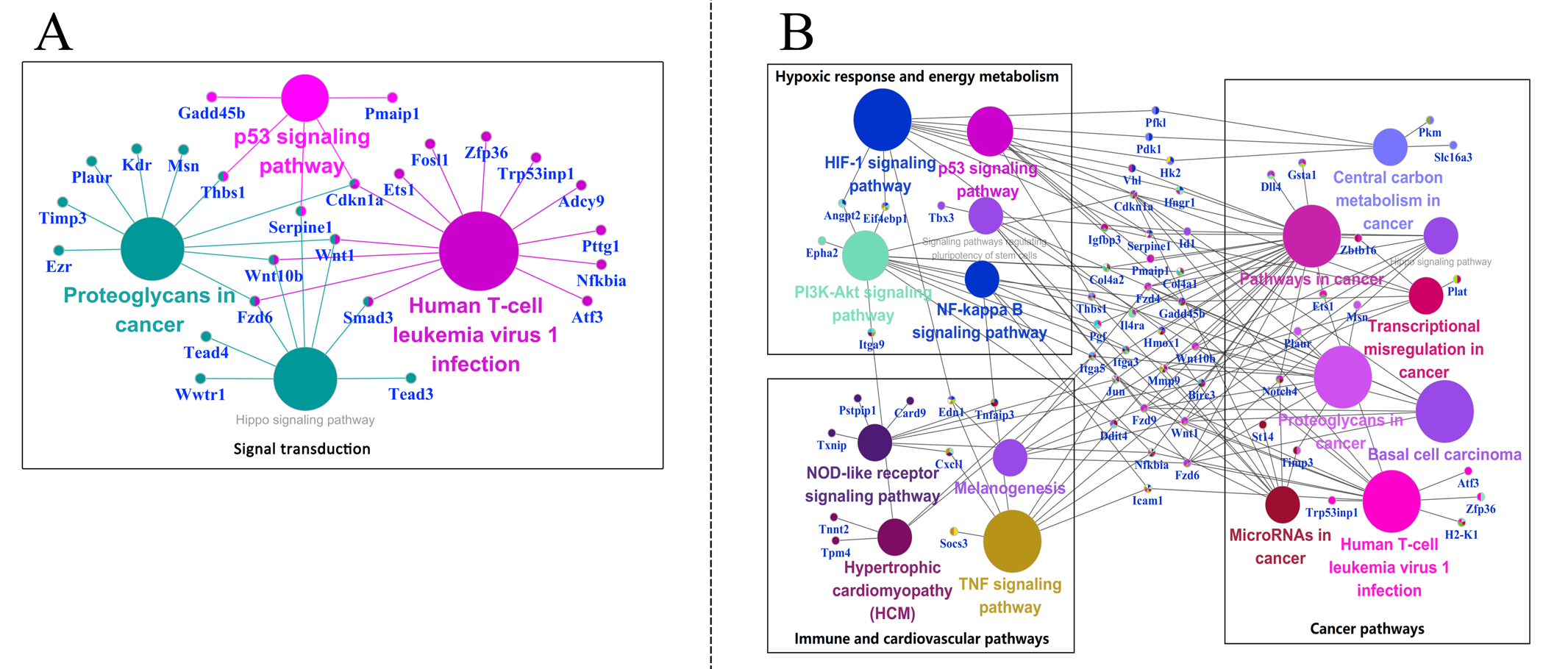

Supplement: Supplementary file 4 — Additional file 4: Figure S3. Gene-pathway networks for DEGs in L. mandarinus (A) and L. brandtii (B) under severe hypoxia determined using the Clue GO tool in Cytoscape. Small and large dots represent DEGs and enriched pathways, respectively. Genes indicated in blue are upregulated. Lines between dots represent connections between the genes and the pathways in the network; genes with more connections are more important in the network. [file 12983_2020_356_MOESM4_ESM.docx]
