# Supplementary material for: Transcriptome analysis of the response provided by Lasiopodomys mandarinus to severe hypoxia includes enhancing DNA repair and damage prevention
Source: Front Zool. 2020 Mar 31;17:9. doi: 10.1186/s12983-020-00356-y (PMC7106638; doi:10.1186/s12983-020-00356-y)

**Figure S4.** Protein interaction network for specific DEGs in *L. brandtii* (A) and *L. mandarinus* (B) brain under hypoxia. Pink squares represent upregulated proteins, and green squares represent downregulated proteins; size of the squares represents the importance of the protein in the network, with bigger squares indicating a greater importance; the thickness of line between the squares represents the strength of the association between two proteins, with a wider line indicating a stronger correlation.


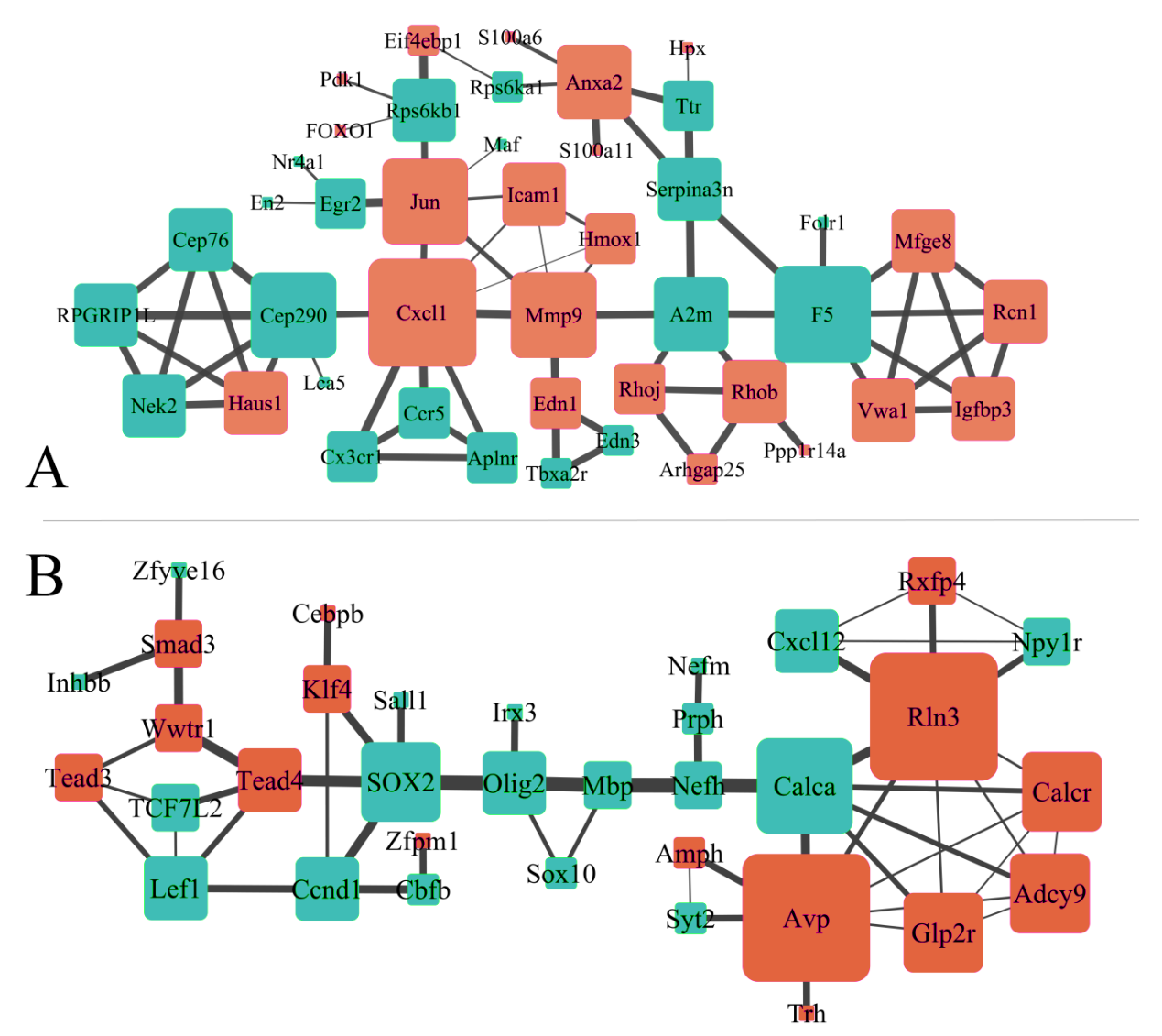

Supplement: Supplementary file 5 — Additional file 5: Figure S4. Protein interaction network for specific DEGs in L. brandtii (A) and L. mandarinus(B) brain under hypoxia. Pink squares represent upregulated proteins, and green squares represent downregulated proteins; size of the squares represents the importance of the protein in the network, with bigger squares indicating a greater importance; the thickness of line between the squares represents the strength of the association between two proteins, with a wider line indicating a stronger correlation. [file 12983_2020_356_MOESM5_ESM.docx]
